# Infra-Abdominal Muscles Activation Brings Benefits to the Pulmonary Function of Patients with Sternal Instability after Cardiac Surgery

**DOI:** 10.21470/1678-9741-2018-0365

**Published:** 2020

**Authors:** Emilia Nozawa, Cristiane Domingues Gonçalves, Patricia Oliva de Almeida, Ludhmila Abrahão Hajjar, Filomena Regina Gomes Galas, Maria Ignêz Zanetti Feltrim

**Affiliations:** 1 Department of Physiotherapy of the Instituto do Coração do Hospital das Clínicas da Faculdade de Medicina da Universidade de São Paulo (InCor/HC-FMUSP), São Paulo, SP, Brazil.; 2 Department of Anesthesia and Surgical Intensive Care of the Instituto do Coração do Hospital das Clínicas da Faculdade de Medicina da Universidade de São Paulo (InCor/HC-FMUSP), São Paulo, SP, Brazil.; 3 Department of Critical Patients of the Instituto do Coração do Hospital das Clínicas da Faculdade de Medicina da Universidade de São Paulo (InCor/HC-FMUSP), São Paulo, SP, Brazil.

**Keywords:** Mediastinitis, Surgical Wound Infection, Sternum, Muscle Strengthening, Exercises

## Abstract

**Objective:**

To compare physical therapy strategies involving abdominal muscle stabilization, with and without upper limb movement, in patients with sternal instability after heart surgery and during in-hospital care.

**Methods:**

This prospective, longitudinal, randomized, and comparative clinical study included 20 patients, which were divided into two groups: ARM, the arm group (n=10), and LEG, the leg group (n=10). The study involved the evaluation of scores of visual analog scales for sternal instability, pain, discomfort, functional impairment, lung function, and maximum inspiratory pressure (MIP) and maximum expiratory pressure (MEP) before and after the interventions. Two protocols consisting of abdominal exercises in both groups with upper limb movements (ARM) and just abdominal activation with leg movements (LEG) were used for three weeks.

**Results:**

There were statistically significant (*P*≤0.01) improvements in pain, discomfort, and functional impairment scores, and in MIP (*P*=0.04) and MEP (*P*≤0.01) after intervention in both groups and just LEG showed improvement in forced vital capacity (*P*=0.043) and forced expiratory volume in one second (*P*=0.011).

**Conclusion:**

Both strategies promoted improvement in pain, discomfort, and functional impairment scores and in the values of inspiratory and expiratory pressures. Perhaps they were influenced by the time and resolution of the infection process, although exercises with upper limb movements seem to be safe in this population. The activation of the infra-abdominal muscles through leg movements seems to bring more benefits to lung function.

**Table:** 

Abbreviations, acronyms & symbols				
ANOVA	= Analysis of variance		InCor/HC-FMUSP	= Instituto do Coração do Hospital das Clínicas da Faculdade de Medicina da Universidade de São Paulo
ARM	= Arm group		LEG	= Leg group
BMI	= Body max index		MEP	= Maximum expiratory pressure
CABG	= Coronary artery bypass grafting		MIP	= Maximum inspiratory pressure
CPB	= Cardiopulmonary bypass		MV	= Mechanical ventilation
CT	= Computed tomography		PEF	= Peak expiratory flow
EF	= Ejection fraction		SPSS	= Statistical Package for the Social Sciences
EuroSCORE	= European System for Cardiac Operative Risk Evaluation		VAC	= Vacuum-assisted closure
FEV_1_	= Forced expiratory volume in one second		VR	= Valve replacement
FVC	= Forced vital capacity			

## INTRODUCTION

Cardiovascular surgery is a common procedure, and the mediastinum is accessed via median sternotomy^[1]^. However, some complications have been described, including mediastinitis, with or without sternal instability. Even with low incidence (0.4 to 5%), it presents high mortality (14% to 47%)^[2,3]^. That is one of the most feared complications by the cardiovascular surgery team, for generating large commitments and functional damages to the patient^[1,4]^. The early recognition and diagnosis of sternal complications is imperative to ensure timely management^[3,5]^. Nowadays, the treatment of mediastinitis consists in drainage, debridement, or complete sternectomy^[6]^. The use of the vacuum-assisted closure (VAC) system and continuous closed mediastinal irrigation with antibiotics has improved the prognosis of these patients^[7,8]^. Sometimes the fibrous union is an end point acceptable process, but patients may experience instability and severe symptoms of pain that affect the quality of life^[9]^.

Movements such as trunk rotation, arm swinging, switching from the sitting to the standing position, and holding objects above shoulder height are limited in these patients, causing pain and discomfort^[10,11]^. These limitations affect lung function, leading to atelectasis and pneumonia secondary to decreased inspiratory capacity^[12]^ and added to inability of the respiratory muscles to generate expiratory flows rates. The sternotomy incision is oriented in the sagittal plane in the same direction of the sacroiliac joint. Contraction of the abdominal muscles, especially the transverse abdominal muscle, exerts “corset-like” forces that decrease sacroiliac joint laxity^[13]^. This effect is also observed in the intercostal muscles that are involved in forced expiration and that pull the sternum downward and inward. Therefore, in cases of sternal instability, the contraction and activation of abdominal muscles associated with active expiration could minimize the excessive movement of the sternum and contribute to its stabilization during trunk and upper limb movements^[14]^.

In clinical practice, it is common to instruct patients to avoid movements involving the upper limbs. The prescription of arm exercises after sternotomy varies among physical therapists even after six weeks of surgery. An Australian study indicated that 95% of the professionals impose movement restrictions, mainly resistive/resistance exercises involving bilateral arm elevation^[10]^. On the basis of the principle that the activation of the abdominal and thoracic cage muscles is the key point to sternal stabilization, we would like to ensure the use of upper limb exercises without causing functional impairment and to investigate if it brings more benefits to an exercise program during in-hospital care.

## Objective

The objective of this study is to compare physical therapy strategies involving abdominal muscle stabilization, with and without upper limb movement, in patients with sternal instability after heart surgery and during in-hospital care.

## METHODS

This prospective, longitudinal, randomized, and comparative clinical study was conducted at the Physiotherapy Department of the Instituto do Coração do Hospital das Clínicas da Faculdade de Medicina da Universidade of São Paulo (InCor/HC-FMUSP). The study was approved by the Research Ethics Committee – CAPPesq of the HC-FMUSP on July 4, 2012, under Protocol No. 51770, and it was registered at www.clinicaltrials.com under NCT02513576.

**Fig. 1:**
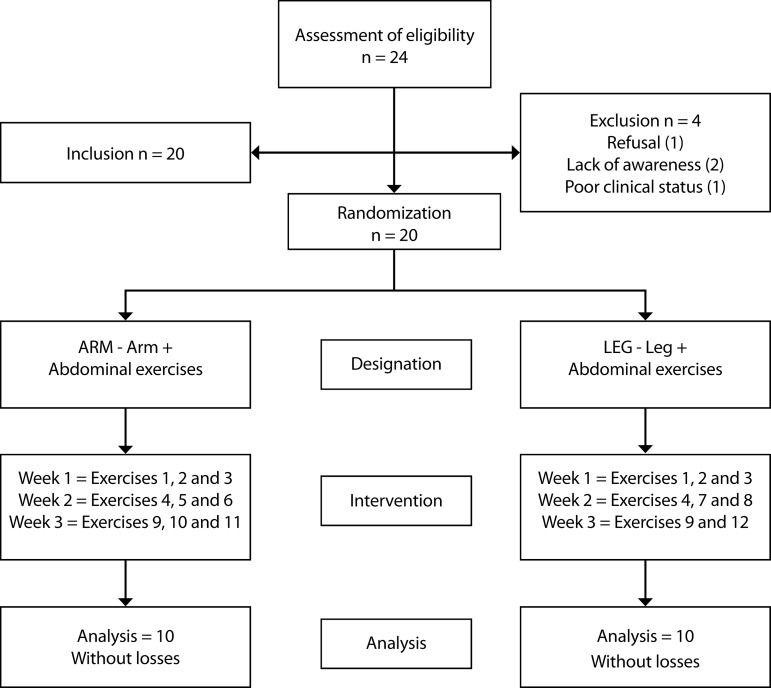
Flow chart of the study.

The study included individuals aged > 18 years, who had been subjected to cardiovascular surgery by median sternotomy. These patients presented sternal instability with or without mediastinitis confirmed by physical examination and chest computed tomography (CT). Patients who were unable to perform the exercise program, had active infection with fever ≥ 38ºC for > 24 hours, and had previous chronic pulmonary disease were excluded. All of the participants signed the informed consent. Patients with wound infection/mediastinitis after sternotomy present a long time of hospitalization in our service, in most cases, more than 15 days with antibiotics and extensive surgical debridement of surgical wound, treatment with negative pressure therapy, and closure of the wound with myocutaneous or fasciocutaneous flaps according to the protocol developed by the InCor/HC-FMUSP^[15]^. All patients who participated in the study used VAC and finalized the intra-hospital protocol. The protocol was started as soon as the instability/wound infection was diagnosed. The study’s flow chart is shown in Figure 1.

### Protocol

**Fig. 2:**
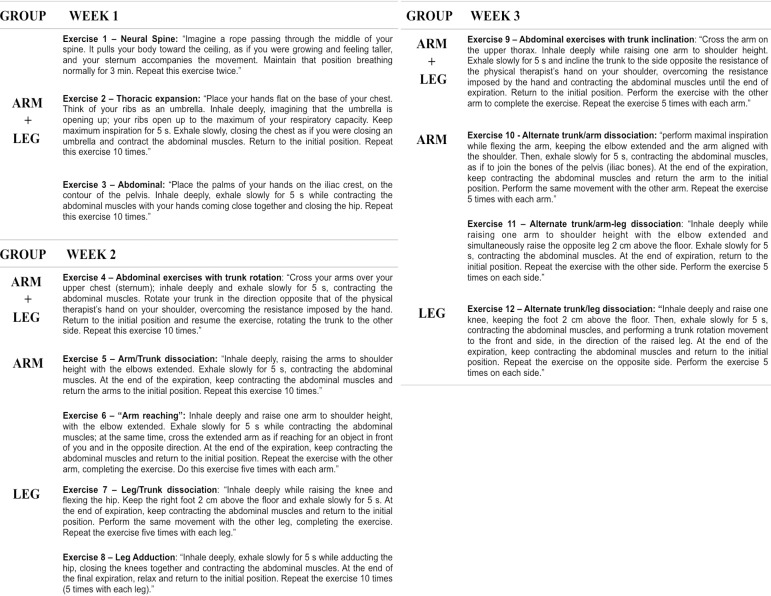
Exercise protocol.

Simple random sampling was used to define the groups: ARM, the arm group, which performed abdominal exercises and movement of upper limbs, and LEG, the leg group, which performed abdominal exercises and movements of lower limbs. All exercises involving lower limbs are only for activation of the infra-abdominal muscles and not for strengthening the leg muscles alone. Considering that movement of lower limbs is related only to the activation of lower abdominal muscles, we suggest that this group can be considered as only abdominal exercises. All the patients followed the institutional exercises program according to benefits related to mobilization^[11,16,17]^, even before and during protocol inclusion, and that routine includes walking for all patients as soon as possible. The exercise session was performed once a day, five times a week, for at least 20 minutes, during three weeks, by the physiotherapist team researchers. The physiotherapist team was trained previously to apply the protocol and collect data and evaluation by the same therapist, who was not blinded. The program of exercises is explained in Figure 2. Briefly, in the first week, both groups performed the same exercises with postural, thoracic expansion and abdominal contraction, and in the second and third weeks, arm movements, for ARM, and movements with legs, for LEG, were included.

Patients could receive analgesia according to the presence of pain, and the drugs of choice were dipyrone (metamizole) and tramadol.

Each exercise was made with maximum voluntary contraction of the abdominal muscles and maintenance of trunk stabilization during expiration, seated on a pattern chair without arm support, and their feet flat on the floor with knees below the hip level, so as to ensure the adequate curvature of the lumbar spine^[11]^.

### Study Variables

Demographic, clinical, and surgical data were collected from the medical records. Data on surgical wound infection, microorganisms, treatment, and use of the VAC system were collected at the start of the study. Chest X-ray and CT images were analyzed to confirm the diagnosis of separation of the sternal edges, mediastinitis, and pleural effusion.

Sternal instability was quantified using the Sternal Instability Scale: 0 (stable sternum), 1 (minimally separated sternum), 2 (partially separated sternum, with a separation smaller than one finger [1.0–1.25 cm]), 3 (marked instability, with a separation larger than one finger), and 4 (complete instability, with a separation > 1.5 fingers)^[5,14]^, previously the VAC intervention.

Pain scale was assessed using the visual analog scale, in which score 0 means absence of pain and 10 corresponds to extreme pain.

Discomfort scale was assessed using the visual analog scale, in which score 0 means absence of discomfort and 10 corresponds to extreme discomfort.

Functional impairment was based on a numerical scale from 0 to 10, as follows: 0–3, did not interfere with activities; 4–6, interfered but did not prevent activities; 7–9, prevented activities; and 10, prevented activities and/or caused lack of control.

The identified daily activities that caused pain and discomfort included coughing, trunk rotation, lying on the side, arm swinging while walk, simulate driving a car, switching from sitting to standing or from lying to sitting, holding an object above shoulder level, and sudden loss of strength during walking.

Lung function was evaluated by spirometry (EasyOne^®^) according to the recommendations of the Sociedade Brasileira de Pneumologia e Tisiologia/American Thoracic Society (2002) and adapted to the Brazilian population, according to Pereira et al. (2007)^[18]^. The forced expiratory volume in one second (FEV_1_), forced vital capacity (FVC), and peak expiratory flow (PEF) were recorded and the obtained values were compared with the expected values^[18,19]^.

Maximum inspiratory pressure (MIP) and maximum expiratory pressure (MEP) were measured using a vacuum manometer (Record®), the measurements were repeated for tree times, and the highest values, excluding the last one, were used^[20]^.

### Statistical Analysis

Data were expressed in terms of percentages and frequencies for categorical variables and in means and standard deviation for continuous variables. To test the normality in the distribution of continuous variables, the Kolmogorov-Smirnov test was used. For comparison between groups, Student's *t*-test and Mann-Whitney U test were used. The paired Student’s *t*-test was used to analyze the variables before and after the intervention and the Wilcoxon test was applied when the hypothesis of normality was rejected. The chi-square (x^2^) test was performed for comparing categorical data. To the intragroup variables, it was used the two-way analysis of variance (ANOVA) for repeated measurements. *P*-value < 0.05 was considered statistically significant. The statistical analysis was performed using the Statistical Package for the Social Sciences (SPSS) software, version 21.0 (SPSS Inc).

## RESULTS

Of the 24 eligible patients, 20 were included in the protocol: 10 patients in ARM and 10 in LEG. The characteristics of the study sample are shown in Table 1. The only variable that presented a statistically significant difference between the groups was the ejection fraction (EF) (%), however, LEG, with a lower mean, presented more variation.

**Table 1:** Patients' demographic, anthropometric, clinical, and surgical characteristics.

Variables	ARM (n=10)	LEG (n=10)	*P*-value*
Age (years)	63±8	58±13	0.120
Gender (male/female)	5/5	7/3	
BMI (kg/cm^2^)	30±5	30±5	0.881
Weight (kg)	81±14	78±11	0.543
Height (cm)	164±8	161±9	0.739
Previous disease			
Hypertension	10 (100%)	10 (100%)	1.000
Diabetes	8 (80%)	4 (40%)	0.068
Smoking (yes)	7 (70%)	7 (70%)	1.000
Acute myocardial infarction	7 (70%)	5 (50%)	0.361
Dyslipidemia	6 (60%)	8 (80%)	0.329
Coronary artery disease	6 (60%)	2 (20%)	0.068
Obesity	4 (40%)	5 (50%)	0.653
Others	3 (30%)	5 (50%)	0.361
Previous surgery	0 (0%)	1 (10%)	0.305
EF (%)	59±6.7	48±15.1	0.029*
Parsonnet score	8.9±7.0	7.5±8.4	0.699
EuroSCORE	1.9±1	3.1±2.14	0.105
Duration of CPB (min)	52±48	63±52	0.853
Time of MV (hours)	14±5	13±7	0.720
Surgery intervention			
CABG	9	9	
CABG + VR	0	1	
Bentall	1	0	

* *P*<0.05

ARM=arm group; BMI=body max index; CABG=coronary artery bypass grafting; CPB=cardio pulmonary bypass; EF=ejection fraction; EuroSCORE=European System for Cardiac Operative Risk Evaluation; LEG=leg group; MV=mechanical ventilation; VR=valve replacement

The patients started the protocol on averaged 18 ± 9.6 days after surgery and the time of hospitalization between the groups to consider the exposure time after surgery and detection of sternal instability is very similar (ARM 17 ± 5.30 days and LEG 17 ± 9.6 days). There was a significant reduction in the pain, task performance, and discomfort scores in both groups after three weeks of the exercises program with *P*<0.01 for all measures (Table 2).

**Table 2:** Pre and post-intervention data of reference of pain, discomfort, and functional impairment in both groups.

	ARM		LEG	
Outcome	Pre	Post	Pre	Post
Pain	3.3±2.6	1.4±1.8*	3.3±2.4	2.0±2.1*
Discomfort	5.4±2.9	2.7±3.4*	4.7±1.8	2.2±1.9*
Functional impairment	4.7±2.2	1.9±2.2*	5.1±2.3	1.5±1.4*

* statistical difference between pre and post-intervention, with *P*=statistical difference < 0.001.

ARM=arm group; LEG= leg group; Pre=pre-intervention; Post=post-intervention

The activities that caused the largest number of complaints of pain and discomfort before the intervention were trunk rotation, lying on the side, coughing, switching from lying to sitting, and holding an object above shoulder height. After the intervention, there was a reduction in the complaints of pain and discomfort related to activities such as lying on the side and coughing. In addition, pain/discomfort related to coughing and to trunk rotation persisted in LEG. Before the exercises, no patient received analgesia.

In the evaluation of the lung function and maximal respiratory pressures, it was used the two-way ANOVA to treat the intragroup and intergroup factors and they are described below (Table 3):

**Table 3:** Pre and post-intervention lung function and maximal respiratory pressures.

Variables	Group	Pre	Post
FVC (L)	ARM LEG*	2.24±0.63 1.95±0.44	2.07±0.63 2.28±0.48
FVC (%)	ARM LEG	63.90±13.03 53.30±13.00	59.40±12.73 61.60±9.06
FEV_1_ (L)	ARM LEG*	1.69±0.58 1.52±0.30	1.61±0.48 1.81±0.32
FEV_1_ (%)	ARM* LEG*	56.90±14.53 51.30±11.43	59.00±15.90 61.30±10.89
PEF (L/s)	ARM LEG	5.63±1.92 4.62±1.28	4.71±2.38 5.22±1.84
PEF (%)	ARM LEG	68.80±25.32 54.40±20.53	60.10±25.32 66.00±23.94
MIP (cmH_2_O)	ARM* LEG*	-73.00±33.09 -77.50±53.50	-93.50±39.72 -94.00±45.81
MEP (cmH_2_O)	ARM* LEG*	75.50±32.18 68.50±28.09	88.50±39.58 82.00±27.30

* statistical difference between pre and post-intervention, with *P*=statistical difference < 0.05.

ARM=arm group; FEV_1_=forced expiratory volume in one second; FVC=forced vital capacity; L=liters; LEG=leg group; PEF=peak expiratory flow; MEP=maximum expiratory pressure; MIP=maximum inspiratory pressure

- FVC: The groups showed a significant difference in behavior (*P*=0.009) and there is an interaction between time and group. Analyzing the groups separately, LEG presents significant increase of FVC from the initial to the final moment (*P*=0.043) and ARM does not show significant changes (*P*=0.104); the groups do not show differences at the initial time (*P*=0.250).
- FVC (%): The groups showed a significant difference in behavior (*P*=0.027), but LEG (*P*=0.063) and ARM (*P*=0.258) do not show differences between the initial and the final moment. However, LEG has more delta variation than ARM (*P*=0.023), which proves the tendency of differences between the initial and the final moment in LEG.
- FEV_1_: The groups showed a significant difference in behavior (*P*=0.003). LEG presents significant increase of FEV_1_ from the initial to the final moment (*P*=0.011) and ARM does not show significant changes (*P*=0.213).
- FEV_1_ (%): The groups did not show any difference in behavior (*P*=0.125) and they did not present significant differences in means at the initial and final moments (*P*=0.766). Both groups present a significant increase in FEV_1_% from the initial to the final moment (*P*=0.024).
- MIP: The groups did not show any difference in behavior (*P*=0.819) and they did not present significant differences in means at the initial and final moments (*P*=0.888). Both groups present a significant increase in MIP from the initial to the final moment (*P*=0.046).
- MEP: The groups did not show any difference in behavior (*P*=0.957) and they did not present significant differences in means at the initial and final moments (*P*=0.626). Both groups present a significant increase in MEP from the initial to the final moment (*P*=0.010).
- PEF: The groups did not show any difference in behavior (*P*=0.073) and they did not present significant differences in means at the initial and final moments (*P*=0.740). Both groups did not present changes of PEF from the initial to the final moment (*P*=0.703).
- PEF (%): The groups did not show any difference in behavior (*P*=0.065) and they did not present significant differences in means at the initial and final moments (*P*=0.655). Both groups did not present changes of PEF% from the initial to the final moment (*P*=0.782).

Regarding the microorganisms found in surgical wound exudates, there was a predominance of *Staphylococcus aureus* (40% of patients in ARM and 60% of patients in LEG), followed by *S. epidermidis* (20% in ARM and 10% in LEG). Other microorganisms, including *Klebisiella*, *Candida albicans*, and *Streptococcus*, were found in 40% of patients in ARM, and *K. pneumoniae carbapenemas*e and *Proteus mirabilis* were found in 20% of patients in LEG. Only one individual in LEG did not have a mediastinal exudate culture, corresponding to 10% of the sample.

## DISCUSSION

An exercises program based in the stabilization of trunk and maximum abdominal muscles contraction seems to be beneficial to patients with sternal instability, independently of using arm movements. More important than that, upper limb exercises appear to have no functional impairment over time. Pain, discomfort, and lung function scores improved significantly in both groups, obviously greatly influenced by the time and resolution of the infection process, but the activation of infra-abdominal muscles, the main objective of lower limb exercises, seems to bring benefits to pulmonary function. Contraction and activation of the abdominal and chest muscles are the main points to trunk stabilization and to reduce the pain and discomfort caused by sacroiliac joint laxity, as described by other studies^[11-13]^.

Myocardial revascularization surgery procedures could involve an internal thoracic artery graft and may decrease the blood supply, leading to hypoxia of sternal bone tissue and regional tissues, which favors the development of infectious microorganisms and mediastinitis. Our finding is that 90% of ARM patients and 80% of LEG patients underwent myocardial revascularization with the use of a thoracic artery graft. Moreover, the previous clinical manifestations, including those related to diabetes mellitus, that changes in the microvasculature, can lead to changes in tissue repair and healing and may predispose to and increase the likelihood of infections^[3]^. Adipose tissue is susceptible to the microbial colonization and proliferation. Several studies reported that patients who smoke, in particular those with chronic obstructive pulmonary disease, are vulnerable to surgical wound infections^[15]^ due lower lung capacity, which impairs tissue oxygenation^[2,5]^. Even though our study exclude chronic pulmonary disease, the effect of active smoking on sternal healing should not be dismissed^[3]^.

Pain, functional impairment, and discomfort may be explained by the excessive motion of the sternal edge, which induces an inflammatory response, and which, in turn, promotes bacterial colonization and infection. Some studies suggest that pain may be related to hypersensitivity reaction to wire, scar entrapped neuralgia, and sternal irritations^[21]^. All study patients underwent treatment of surgical wound infections using the VAC system. This treatment tends to be more painful than only antibiotic therapy because of increased invasiveness and the need for debridement of the surgical incision^[7,8]^. However, the treatment used in our sample decreased significantly the overall mortality and the rates of recurrence and readmission^[15]^.

The pain and discomfort reported by the participants in the surgical wound area may also be associated with the magnitude of sternal separation^[12,13]^. In addition, pain/discomfort related to coughing and to trunk rotation persisted in LEG, despite the improvement in other activities. Avoiding movement in order to avoid pain deconditions muscle, whereas exercise improves muscle function^[13]^, and this perhaps justify the permanence of pain in activities that demand greater strength for thoracic stability in the group without training of upper limb muscles.

Prolonged surgical time, hemorrhage, high risk score, and ineffective antibiotic therapy increase the prevalence of mediastinitis. Therefore, a reduction in lung volume and capacity, a decrease in lung compliance, and changes in gas exchange favor alveolar collapse caused by hypoventilation, such as atelectasis. These changes were observed in the present study, wherein the values of the lung function (FVC, FEV_1_, and PEF) were lower than expected in both groups^[18,19]^. This finding may be explained by the surgical procedure, the surgical access via median sternotomy, the number of required repeat surgeries, and wound infection. All these factors lead to a decrease in the compliance of the thoracic cage, resulting in a reduction in lung volumes and capacities.

The values of MIP/MEP were altered in both groups, which indicated respiratory muscle weakness^[20,22,23]^. After the intervention, there was a significant increase in the mean values of MIP (*P*=0.04) and MEP (*P*<0.01) in both groups, considering the absolute value that represents the pressure developed by the respiratory muscles. A previous study indicated that respiratory muscle performance was decreased significantly in the postoperative period and that this reduction was related to the presence of pain, and that analgesics and heart surgery affected muscle function^[23]^. The reduction in MEP may be associated with the changes in respiratory muscle function combined with sternal instability caused by sternum bone section, which affects the adequate activation of the abdominal muscles during physical activities and leads to thoracic/abdominal imbalance and a decrease in inspiratory and expiratory muscle strength.

About FVC, LEG presents significant increase from the initial to the final moment (*P*=0.043), ARM does not show significant changes (*P*=0.104), and LEG have more delta variation than ARM (*P*=0.023), which proves the tendency of difference between the initial and final moments in LEG and this group presents significant increase of FEV_1_ from the initial to the final moment (*P*=0.011). Therefore, the abdominal exercises performed by LEG may have provided a greater abdominal stabilization, with the best activation of infra-abdominal muscles and improvement of body awareness and increased recruitment of respiratory muscles, than those performed by ARM.

The cardiac function related to EF (%) presented a statistical difference between the groups. However, the risk scores showed no difference and the lowest mean EF value of this is study is 48% in LEG. The literature considered EF ≤ 40% to estimate risk of mediastinitis^[2]^, and the preserved EF is usually defined as an EF ≥ 50%, although some studies have used a limit as low as 40%^[24]^. May be this suggests low influence of EF (%) on the outcomes of the groups. Moreover, although the European System for Cardiac Operative Risk Evaluation (EuroSCORE) did not showed statistical difference, there is a clinically significant difference in both groups, with score 3 in LEG meaning a moderate risk, LEG could possibly have a faster recovery after the protocol than ARM. Several studies have shown that patients with left ventricular dysfunction have greater improvements with postoperative rehabilitation compared to normal left ventricular function subjects.

Surgical wound infection is a severe postoperative complication and leads to prolonged in-hospital stay, increased hospital costs, and high rates of patient morbidity and mortality^[15]^. Regarding the microorganisms identified in cultures of the surgical wounds, the incidence of *S. aureus* both in ARM (40%) and LEG (60%) was higher than that of other microbial agents, which confirms the results reported in the literature^[6,7]^. However, the cultures of surgical wound exudates may be negative for microorganisms in some cases because of the prophylactic use of antibiotics^[4]^.

The limitations of this study were: 1) the low incidence of sternal instability/mediastinitis that makes this a small sample; 2) the use of the VAC system that did not allow the adequate palpation of the bone structure during the pre-intervention evaluation; 3) the study was not conducted in a blind manner.

Although statistical analysis had demonstrated a reduction in the pain and discomfort scores in both groups, it did not only attribute those to abdominal exercises. Since inflammation and pain usually decrease over time, this improvement could be a natural response to postoperative recovery.

## CONCLUSION

Both strategies promoted improvement in pain, discomfort, and functional impairment scores, perhaps influenced by the time and resolution of the infection process, however, the use of protocols with upper limb movements seems to be safe and, more than that, the activation of the infra-abdominal muscles through leg movements seems to bring more benefits to lung function. We emphasize the need for larger studies on the rehabilitation of patients with sternal instability during in-hospital care.

**Table:** 

Author's roles & responsibilities	
EN	Substantial contributions to the conception or design of the work; or the acquisition, analysis, or interpretation of data for the work; drafting the work or revising it critically for important intellectual content; final approval of the version to be published
CDG	Substantial contributions to the conception or design of the work; or the acquisition, analysis, or interpretation of data for the work; drafting the work or revising it critically for important intellectual content; final approval of the version to be published
POA	Substantial contributions to the conception or design of the work; or the acquisition, analysis, or interpretation of data for the work; final approval of the version to be published
LAH	Substantial contributions to the conception or design of the work; or the acquisition, analysis, or interpretation of data for the work; final approval of the version to be published
FRGG	Substantial contributions to the conception or design of the work; or the acquisition, analysis, or interpretation of data for the work; final approval of the version to be published
MIZF	Substantial contributions to the conception or design of the work; or the acquisition, analysis, or interpretation of data for the work; drafting the work or revising it critically for important intellectual content; final approval of the version to be published
